# Multiple coping strategies maintain stability of a small mammal population in a resource‐restricted environment

**DOI:** 10.1002/ece3.7997

**Published:** 2021-08-16

**Authors:** Anne Y. Polyakov, William D. Tietje, Arjun Srivathsa, Virginie Rolland, James E. Hines, Madan K. Oli

**Affiliations:** ^1^ Department of Environmental Science, Policy, and Management University of California Berkeley CA USA; ^2^ School of Natural Resources and Environment University of Florida Gainesville FL USA; ^3^ Department of Biological Sciences Arkansas State University Jonesboro AR USA; ^4^ US Geological Survey, Patuxent Wildlife Research Center Laurel MD USA; ^5^ Department of Wildlife Ecology and Conservation University of Florida Gainesville FL USA

**Keywords:** capture–mark–recapture, climate change, climatic effects, resilience, small mammal population dynamics

## Abstract

In semi‐arid environments, aperiodic rainfall pulses determine plant production and resource availability for higher trophic levels, creating strong bottom‐up regulation. The influence of climatic factors on population vital rates often shapes the dynamics of small mammal populations in such resource‐restricted environments. Using a 21‐year biannual capture–recapture dataset (1993 to 2014), we examined the impacts of climatic factors on the population dynamics of the brush mouse (*Peromyscus boylii*) in semi‐arid oak woodland of coastal‐central California. We applied Pradel's temporal symmetry model to estimate capture probability (*p*), apparent survival (*φ*), recruitment (*f*), and realized population growth rate (*λ*) of the brush mouse and examined the effects of temperature, rainfall, and El Niño on these demographic parameters. The population was stable during the study period with a monthly realized population growth rate of 0.993 ± *SE* 0.032, but growth varied over time from 0.680 ± 0.054 to 1.450 ± 0.083. Monthly survival estimates averaged 0.789 ± 0.005 and monthly recruitment estimates averaged 0.175 ± 0.038. Survival probability and realized population growth rate were positively correlated with rainfall and negatively correlated with temperature. In contrast, recruitment was negatively correlated with rainfall and positively correlated with temperature. Brush mice maintained their population through multiple coping strategies, with high recruitment during warmer and drier periods and higher survival during cooler and wetter conditions. Although climatic change in coastal‐central California will likely favor recruitment over survival, varying strategies may serve as a mechanism by which brush mice maintain resilience in the face of climate change. Our results indicate that rainfall and temperature are both important drivers of brush mouse population dynamics and will play a significant role in predicting the future viability of brush mice under a changing climate.

## Graphical abstract

We show that brush mice maintain population stability through multiple coping strategies, investing in recruitment during warmer, drier periods and in survival during cooler, wetter conditions. Our work is significant and timely because it not only allows us to better predict small mammal population dynamics, but also promotes a deeper understanding of the mechanisms of species resilience with climate change. This research also helps inform conservation and management goals for small mammal populations, which are especially important as small mammals play significant roles in ecosystem food webs and act as reservoir hosts for viral infections.

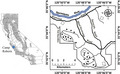

## 1 INTRODUCTION

Changes in abundance of small mammal populations can result from complex interactions among multiple factors, such as climate, plant production for food supply, vegetative cover for refuge and nesting, predation, and competition (Oli & Dobson, 2003). The debate over the relative roles of endogenous (e.g., competition and predation) and exogenous (e.g., temperature and rainfall) factors in population dynamics has resulted in a general agreement that both influence population fluctuations (Turchin, 2003). However, some studies in semi‐arid systems show that exogenous factors outweigh endogenous factors in driving small mammal population dynamics and are key to understanding fluctuations (Gutiérrez et al., 2010; Previtali et al., 2009), particularly for evaluating the persistence of populations on the margins of a species’ distribution (Gillespie et al., 2008).

Semi‐arid landscapes are highly variable in seasonal and annual rainfall patterns, typically with hot, dry summers and cool, wet winters. These systems are also resource‐restricted, exhibiting pulse‐like patterns of annual rainfall (averaging 25–50 cm) and large seasonal fluctuations, with nearly all rainfall occurring in the fall and winter months (Peel et al., 2007). Fluctuations in rainfall may also be erratic, with some years of high and above‐average precipitation (due to the El Niño effect in some areas), and other years of very little precipitation, at times leading to severe drought (Previtali et al., 2009). In such systems, precipitation typically drives plant productivity, primarily through seed production and foliage growth (Brown & Ernest, 2002; Heske et al., 1994; Lima et al., 2002; Meserve et al., 2003).

Many studies show that dramatic changes in precipitation have strong effects on population dynamics of small mammals, with rainfall pulses driving rodent dynamics indirectly through primary production, such as food availability and cover (Brown & Ernest, 2002; Heske et al., 1994; Knapp et al., 2008; Meserve et al., 2003; Yates et al., 2002). Conversely, drought can have a detrimental effect on rodent population dynamics by reducing plant productivity (Brown & Ernest, 2002; Meserve et al., 2003), sometimes leading to population collapse (Facka et al., 2010). However, we know relatively little about the effect of temperature on small mammal population dynamics in semi‐arid climates, although some studies suggest that the effects of temperature are seasonal, showing a negative correlation with survival and recruitment in summer and a positive correlation in winter (Luis et al., 2010; Myers et al., 1985).

**FIGURE 1:**
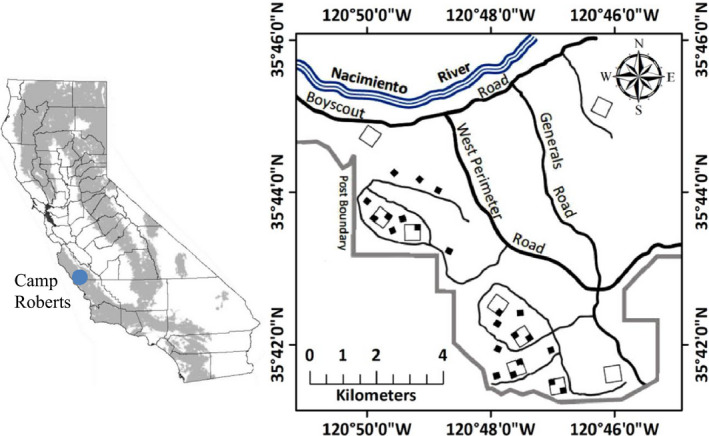
Map of the study area. Trapping was carried out on nine 5.8‐ha 17 × 17 trapping grids (open, larger squares) from 1993 to 1996 and on twenty‐two 1.1‐ha 8 × 8 trapping grids (solid, smaller squares) from 1997 to 2014. Inset map of California is adapted from the California wildlife habitat relationships system range maps (California Department of Fish and Wildlife, 2014). Location of the study area (solid circle) and the distribution of the brush mouse (*Peromyscus boylii*; gray shading) in California, USA

Due to their short life cycles, small mammals serve as ideal systems for long‐term, multi‐generational studies. Short life spans and fast reproduction also translate to quick responses to changes in climatic conditions (Previtali et al., 2009). The brush mouse (*Peromyscus boylii*) has a wide distribution in the United States, occurring throughout much of the southwest and most of California (Figure 1), where it is typically found in mature chaparral, oak woodland, and hardwood‐conifer communities (Baker, 1968). As its name suggests, the brush mouse prefers significant amounts of tree cover, dense and shrubby vegetation, rock cover, and logs, which are important habitat structures that provide shelter from weather and predators, as well as nesting sites (Bradley & Schmidly, 1999; Brehme et al., 2011; Gottesman et al., 2004; Kuenzi et al., 1999; Morrison et al., 2002). The brush mouse plays an important role in ecosystem function as a key prey species for the federally threatened Mexican spotted owl, (*Strix occidentalis lucida*; Boyett, 2001). In the southwestern United States, the brush mouse is also a reservoir host for hantavirus (Abbott et al., 1999), making studies of population dynamics important for predicting rates of disease prevalence and spread. Anthropogenic impacts on brush mouse habitat include exurban development and increasing wildfire intensity (Brehme et al., 2011). From our knowledge, no study has provided estimates of apparent survival, recruitment, and realized population growth rate of the brush mouse, which are essential for understanding population dynamics of this species.

Our goal was to examine the effects of temperature, precipitation, and El Niño on the population dynamics of the brush mouse in a coastal‐central California mixed‐oak woodland. We applied temporal symmetry capture–mark–recapture (CMR) models (Nichols et al., 2000; Pradel, 1996; Williams et al., 2002) to a long‐term (1993–2014) dataset to (a) estimate overall and seasonal patterns of capture probability, apparent survival, recruitment, and realized growth rate of the brush mouse, (b) determine the relative contributions of survival and recruitment to population growth rate, and (c) explore the role of rainfall and temperature in explaining variations in population vital rates.

We expected that brush mouse survival, recruitment, and realized population growth rate would exhibit seasonal fluctuations in response to the strongly seasonal patterns of rainfall at our study site. Specifically, because water is a limiting resource in semi‐arid environments (Gutiérrez et al., 2010; Previtali et al., 2009) and plant cover is an important habitat attribute for the brush mouse, we predicted that brush mouse population parameters would be positively influenced by rainfall. We expected that warmer temperatures would negatively influence brush mouse vital rates due to higher energetic costs associated with foraging (Chen et al., 2015), although warmer temperatures could positively affect recruitment by creating more favorable conditions during winter for reproduction (Andreo et al., 2009). We expected brush mouse vital rates to exhibit multiannual fluctuations corresponding to El Niño, which affects rainfall patterns in California. Finally, because of the fast life history of the species (Heppell et al., 2000; Oli, 2004; Oli & Dobson, 2003; Oli et al., 2005), we predicted that the population growth rate of the brush mouse would be influenced more by recruitment than by survival. Our results provide information on potential impacts of climate change on brush mouse population ecology and provide important management information for the species.

## 2 METHODS

### 2.1 Study area

We conducted the study at the Camp Roberts National Guard Post, a 17,000‐ha military facility located in coastal‐central California (Figure 1). Our study area was located in the backcountry of the Post, a roughly 4,000‐ha matrix of undisturbed grassland, chaparral, and woodland. Climate of the study area is Mediterranean, with cool, wet winters and warm, and dry summers. Annual rainfall is highly variable and is influenced by El Niño‐La Niña oscillations. The long‐term annual average rainfall (1960–2019) at the study area was 37.4 cm (National Oceanic & Atmospheric Administration, 2016), with more than 95% of the rainfall typically falling between October and April. During the study, mean monthly rainfall during May to September was 0.33 cm (range: 0–4.75 cm, standard deviation: 5.36 cm) compared to a monthly mean of 5.26 cm during October to April (range: 0–31.27 cm, standard deviation: 5.24 cm). The study area consisted of pure stands of blue oak (*Quercus douglassii*) on the more xeric sites, and a mix of blue oak and coast live oak (*Q*. *agrifolia*) on more mesic sites. The more mesic areas usually included a shrub layer of up to 35% cover (Tietje et al., 1997) and a ground layer of introduced Mediterranean annual grasses (*Avena* spp.) and forbs.

### 2.2 Field methods

In summer 1993, we laid out nine square 5.8‐ha plots in areas with at least 60% tree canopy cover. On each plot, we established with compass and tape a 17 × 17 grid with 15‐m intersections. We marked each of the 289 intersections per plot with a stake and a survey flag with alphanumeric grid location. We trapped small mammals at each intersection in May and October. From October 1993 to October 1996, we trapped for 5 nights each session for 7 trapping sessions (9 plots with 289 traps * 5 nights * 7 sessions = 91,035 trap nights). To increase the number of sampling grids on the study area, in winter of 1997 we established twelve 1.1‐ha plots and set up an 8 × 8 trapping grid with 15‐m intersections on each plot (Figure 1). Starting in May 1997, we trapped on these 12 plots and on one 8 × 8 corner (1.1 ha) or on two diagonal corners of six of the 5.8‐ha plots. From May 1997 until May 2013, we sampled exclusively on these twenty‐two 8 × 8 sampling grids for a total of 139,392 trap nights (22 plots with 8 × 8 traps * 3 nights * 33 sessions). In October 2013, we trapped on 21 of the 22 plots for a total of 4,032 trap nights. Finally, in May 2014, we trapped on 9 of the 22 plots (1,728 trap nights), for a total of 236,187 trap nights during the 21 years of study from 1993 to 2014. We will refer to the periods between trapping sessions as “season”, either a summer season (5‐month warm, dry period from May to September that preceded the October trapping session) or a winter season (the 7‐month cool, wet period from October to April that preceded the May trapping session).

During each May and October sampling session, we placed one Sherman live trap (7.6 × 8.9 × 30 cm; H.B. Sherman Traps, Inc., Tallahassee, Florida) within 2 m of each grid intersection. To insulate trapped animals from overnight cold and from the heating of the interior of the trap by early morning sunshine, traps were placed in shade and covered with grass and other litter from the vicinity of the trap. We baited traps with a mixture of rolled oats, corn, and barley laced with molasses. On initial capture, we placed a laser‐etched Monel 1005‐1L1 animal tag (National Band and Tag Co., Newport, Kentucky) in the animal's right ear and recorded trap location, tag number, species, sex, and age (juvenile if ≥25% gray pelage, or adult). A ripped ear, potentially caused by a lost tag, was almost never observed. We released animals at site of capture. All handling of animals followed the guidelines of the University of California, Berkeley, Institutional Animal Care and Use Committee (UCB Permit # R‐166). Trapping also met the guidelines of the American Society of Mammalogists (Sikes, 2016).

### 2.3 Capture–mark–recapture analysis

We used Pradel’s (1996) temporal symmetry model to estimate recapture probability (*p*), apparent survival (*φ*), recruitment (*f*), and realized population growth rate (*λ*) (Table 1). Apparent survival includes losses from both mortality of individuals, as well as permanent emigration of individuals out of the study area. Recruitment includes gains of new individuals from both reproduction and immigration of individuals into the study area. We used only adult individuals; we did not include juveniles in the analyses. First, we fitted a series of base models where we allowed *p, φ*, and *λ* to be affected by time (time refers to sampling occasions, where October 1993 is sampling occasion 1 and May 2014 is sampling occasion 42), year, season, and sex, and by the additive and interactive effects of these variables (Table 2a); we estimated model parameters using the most parsimonious model in the set (based on Akaike's information criterion corrected for small sample size AICc; Burnham & Anderson, 2002; Williams et al., 2002). After selecting the base model for each parameter, we tested for the effect of individual climatic covariates on that parameter using the base model. This second set of models allowed the model parameters to be affected by sea surface temperature anomaly (a measure of El Niño Southern Oscillation), temperature, and rainfall (Table 2c). We used a similar modeling approach to test for the effects of climatic covariates on *f* using the *φ*–*f* parameterization of Pradel's model (Williams et al., 2002).

**FIGURE 2:**
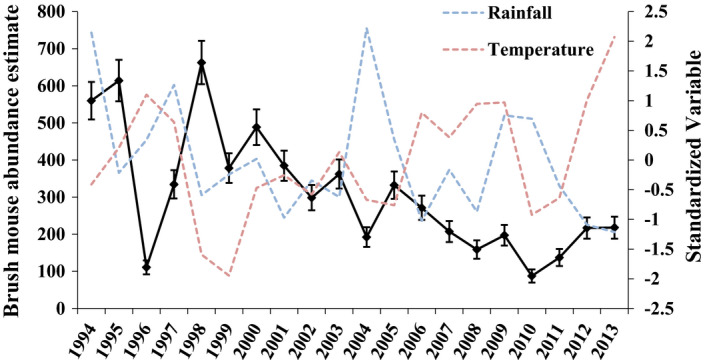
Abundance estimates (± *SE*) of brush mice for summer and winter sessions on nine 5.8‐ha study plots (17 × 17 sampling grid) during October 1993 to October 1996 and on 22 1.1‐ha study plots (8 × 8 sampling grid) during May 1997 to May 2014 at Camp Roberts, CA. The blue and orange dotted lines represent a standardized rainfall and temperature index

Because we altered the area sampled in October 1997 (see Figure 1), we ran the *φ*–*λ* parameterization of Pradel's model to obtain *λ* estimates for winter 1998 to summer 2012. In addition, because the first estimate of *λ* is typically inestimable in time‐specific models, we omitted the first estimate of *λ*. We also tested for the effect of the sampling area change that occurred in October 1997 by including an additive effect of sampling area on model parameters. We included this additive effect of sampling area in the best model without covariates (the top model in Table 2a) and reported the difference in AICc for models with and without the additive effect of sampling area (Table 2b). We determined the relative contribution of *φ* and *f* to *λ* by calculating the proportional contribution of the seniority parameter *γ* (Nichols & Hines, 2002; Schorr, 2012). If *γ* > 0.5, *φ* influences *λ* more strongly than *f*. We did not consider spatial grid‐to‐grid variation and conducted a single analysis that combined data from all grids into one large population. Finally, we estimated population size by using the super‐population (or POPAN) model, which is a reparameterization of the Jolly–Seber model (Arnason & Schwarz, 1995; Schwarz & Arnason, 1996; Williams et al., 2002; Figure 2).

We performed all analyses with the program MARK (White & Burnham, 1999) v. 6.2 through RMark (Laake, 2013) in program R v. 2.2.0 (R Core Team, 2019). We determined the effect of climatic covariates by comparing AICc for models with and without a covariate, based on 95% confidence intervals for the slope parameter defining the relationship between a demographic parameter and the covariate(s).

### 2.4 Climatic covariates

We extracted average daily temperature and precipitation for 1993–2015 from the Paso Robles City National Climatic Data Center weather station (National Oceanic & Atmospheric Administration, 2016), located in Paso Robles, California, approximately 11 km southeast of the study area. We explored whether climatic conditions during the current season or the previous season (one‐lag) affected brush mouse vital rates (survival, recruitment, and growth). For example, for a May 2008 trapping session, the current season would indicate the climatic conditions from October 2007 to April 2008 (a winter season), while the previous season would indicate climatic conditions from May 2007 to September 2007 (a summer season). We used the following climatic variables: average temperature (temp_avg), coefficient of variation (CV) of temperature (temp_cv), CV of temperature with a one‐season lag (temp_cv_onelag), total seasonal rainfall (rain_sum), total seasonal rainfall with a one‐season lag (rain_sum_onelag), CV of rainfall (rain_cv), and CV of rainfall with a one‐season lag (rain_cv_onelag). We reported the estimates of slope parameters (*β*) based on the most parsimonious model that included a given covariate (temperature and rain) for each vital rate (survival, recruitment, and growth), regardless of model structure for other demographic rates.

To examine effects of the El Niño Southern Oscillation (ENSO) on small mammal population dynamics, we used the Oceanic Niño Index (ONI), the standard used by the National Oceanic and Atmospheric Administration to identify El Niño and La Niña events in the Pacific Ocean. An El Niño or La Niña is characterized by five consecutive 3‐month sea surface temperatures means above (for El Niño) or below (for La Niña) a threshold of +0.5°C (−0.5°C), measured above the equatorial Pacific. We extracted ONI values from the National Oceanic and Atmospheric Administration National Weather Service Climate Prediction Center (http://www.cpc.noaa.gov/products/analysis_monitoring/ensostuff/ensoyears.shtml) and information pertaining to the ENSO cycle from the National Oceanic and Atmospheric Administration's Climate website (https://www.climate.gov/enso).

## 3 RESULTS

During the study (1993–2014), we captured 3,258 (1,634 female and 1,624 male) brush mice 6,351 times. The highest estimated abundance of brush mice was 568 in fall 1993, and the lowest abundance of mice was 11 in fall 1996 (Figure 2). Most mice were trapped for one year or less; however, we trapped several (28) brush mice for 1.5 to 2.5 consecutive years. One female was trapped for 3.5 years. For models without covariate effects (Table 2b), the model without an additive effect of sampling area was favored over models with an additive effect of sampling area (ΔAICc = 22.77) for both survival and recruitment, suggesting that the change in sampling area had no discernible effect on survival or recruitment.

**TABLE 1:** Overall, sex‐specific, and season‐specific estimates of monthly apparent survival (*φ*), capture probability (*p*), monthly recruitment (*f*), and realized monthly growth rate (*λ*) without covariate effects estimated using Pradel's model fitted to brush mice (*Peromyscus boylii*) capture–mark–recapture data

	*p*	*φ*	*f*	*λ*
Female	0.700 ± 0.055	0.815 ± 0.005	0.177 ± 0.038	0.993 ± 0.033
Male	0.468 ± 0.046	0.815 ± 0.005	0.173 ± 0.038	0.993 ± 0.033
Winter	0.526 ± 0.048	0.848 ± 0.009	0.137 ± 0.009	1.009 ± 0.010
Summer	0.641 ± 0.053	0.764 ± 0.011	0.240 ± 0.014	0.973 ± 0.014
Overall	0.584 ± 0.051	0.789 ± 0.005	0.175 ± 0.038	0.993 ± 0.032

Note

Overall, sex‐specific and season‐specific estimates of *φ* were based on the third and fifth‐ranked model from Table 2a. Sex‐specific and season‐specific estimates of *f* were based on the first, fourth, and twenty‐fifth ranked model from Table 2a. Sex‐specific and season‐specific estimates of *λ* were based on the fifth and twenty‐third ranked model from Table S1.

### 3.1 Demographic parameters without covariate effects

**FIGURE 3:**
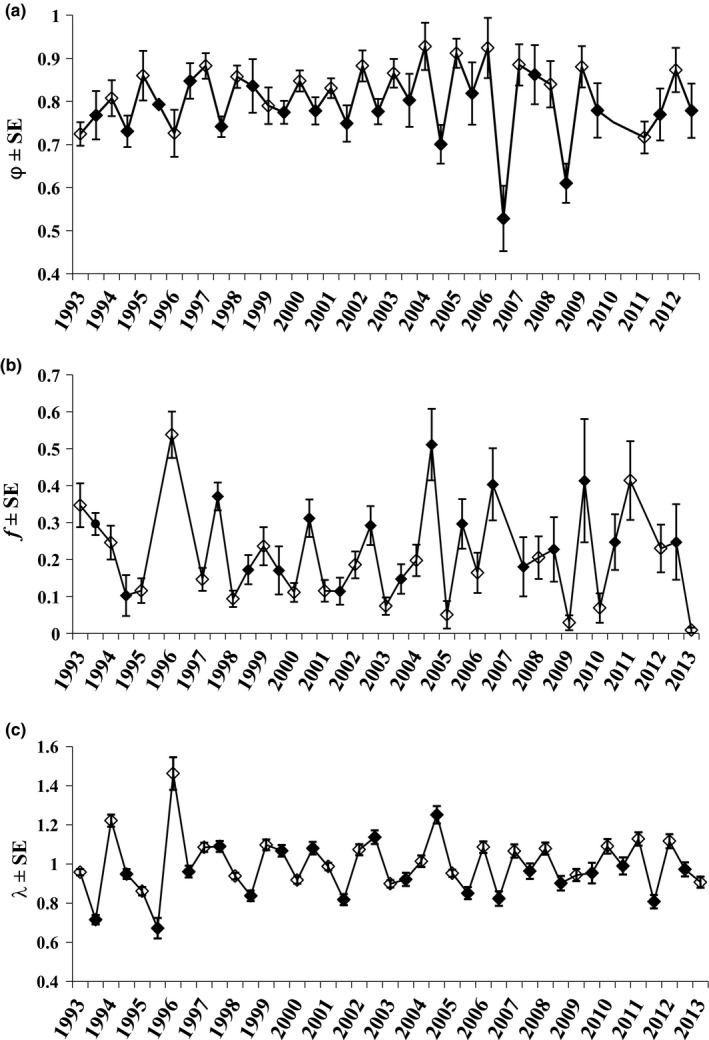
(a–c) Estimates of (a) monthly apparent survival (*φ*), (b) recruitment rate (*f*), and (c) realized population growth rate (*λ*) of brush mice (*Peromyscus boylii*) at Camp Roberts, California, from winter 1993 to winter 2013 for *φ*, *f*, and *λ* based on the most parsimonious model (Table 2a, c). The first estimate for *λ* is excluded because it is typically inestimable in time‐specific models. Summer season estimates are indicated by a solid black point, and winter seasons are indicated by an open point

Pradel's models indicated some level of temporal variation in all demographic parameters: *p*, *φ*, *f*, and *λ*. Average *p* was higher for females than for males and higher in summer than in winter for both sexes (Table 1). Overall monthly *φ* was 0.789 ± 0.005 (annual *φ* was 0.789^12^ = 0.058), and seasonal *φ* was higher in winter (0.848 ± 0.009) than in summer (0.764 ± 0.011; Table 1). Estimates for seasonal *φ* ranged from 0.620 ± 0.030 (summer 1994) to 0.946 ± 0.034 (winter 2005) and showed only small fluctuations over the study period, except when *φ* decreased from 0.924 (winter 2006) to 0.528 (summer 2007), a 57% decrease (Figure 3). Overall monthly *f* was 0.175 ± 0.038 and varied substantially over time, ranging from 0.029 ± 0.047 to 0.538 ± 0.063. Seasonal recruitment was considerably higher in summer (0.240 ± 0.014) than in winter (0.137 ± 0.009; Table 1). Time‐specific *f* parameters were inestimable (confidence intervals from 0 to 1) during three seasons (summer 1996, summer 1997, and winter 2007; Figure 3).

Our study population was stable during the 21 years of study (*λ* = 0.993 ± 0.032, *φ*–*f* parameterization; Table 1), but estimated *λ* exhibited strong time‐variation, particularly during winter 1993 to summer 1997. The largest variation in estimated *λ* occurred when growth rate increased from its lowest point during the 21‐year period, 0.672 ± 0.052 in summer 1996, to 1.46 ± 0.08 in winter 1996––a 118% increase (Figure 3). The most parsimonious model (based on AICc) included an additive effect of sex and season for *p*, and a time effect on *f, φ*, and *λ* (Table 2a). Estimates for the proportional contribution parameter (*γ*) ranged from 0.515 to 0.969, with a mean of 0.794 (*SD* = 0.097).

**TABLE 2:** (a–c) Model selection results for Pradel's model fitted to capture–mark–recapture data for brush mice (*Peromyscus boylii*), testing for the effect of time (trapping session), year, season (winter or summer), and sex (male or female)

Model	*K*	ΔAICc	Weight
(a)			
*φ*(~time) *p*(~season + sex) *f*(~time)	85	0	0.441
*φ*(~time) *p*(~season * sex) *f*(~time)	86	1.81	0.179
*φ*(~time + sex) *p*(~season + sex) *f*(~time)	86	2.18	0.148
*φ*(~time + sex) *p*(~time) *f*(~time)	86	3.77	0.067
*φ*(~season) *p*(~season * sex) *f*(~time)	87	3.85	0.064
(b)			
*φ*(~time) *p*(~season + sex) *f*(~time)	85	0	0.99
*φ*(~time + sampling) *p*(~season + sex) *f*(~time + sampling)	87	22.77	1.14 × 10^–5^
*φ*(~time + sampling) *p*(~season + sex) *f*(~time)	86	38.03	5.53 × 10^–9^
*φ*(~time) *p*(~season + sex) *f*(~time + sampling)	86	41.56	9.43 × 10^–10^
(c)			
*φ*(~rain_cv) *p*(~season + sex) *f*(~temp_avg)	46	0	0.921
*φ*(~rain_cv) *p*(~season + sex) *f*(~rain_sum)	46	5.98	0.046
*φ*(~rain_sum_onelag) *p*(~season + sex) *f*(~rain_cv_onelag)	46	7.17	0.026
*φ*(~rain_sum_onelag) *p*(~season + sex) *f*(~temp_avg)	46	12.82	0.002
*φ*(~temp_avg) *p*(~season + sex) *f*(~temp_avg)	46	13.06	0.001

Note

Parameters are as follows: *φ* = apparent survival probability; *p* = capture probability; and *f* = rate of recruitment. The number of parameters (*K*), difference in Akaike's information criterion corrected for small sample size between a given model and the top‐ranked model (ΔAICc), and the relative model weight are also given. The five best‐supported models are presented. A plus sign (+) indicates additive, and an asterisk (*) indicates both additive and interactive effects of the covariates involved. (a) Models for monthly apparent survival (*φ*) and recruitment (*f*) rates without covariate effects. (b) Models testing for the effect of sampling area on *φ* and *f*. (c) Models testing for the singular effect of climatic covariates on *φ* and *f*.

### 3.2 Individual climatic covariate effects on demographic parameters

**FIGURE 4:**
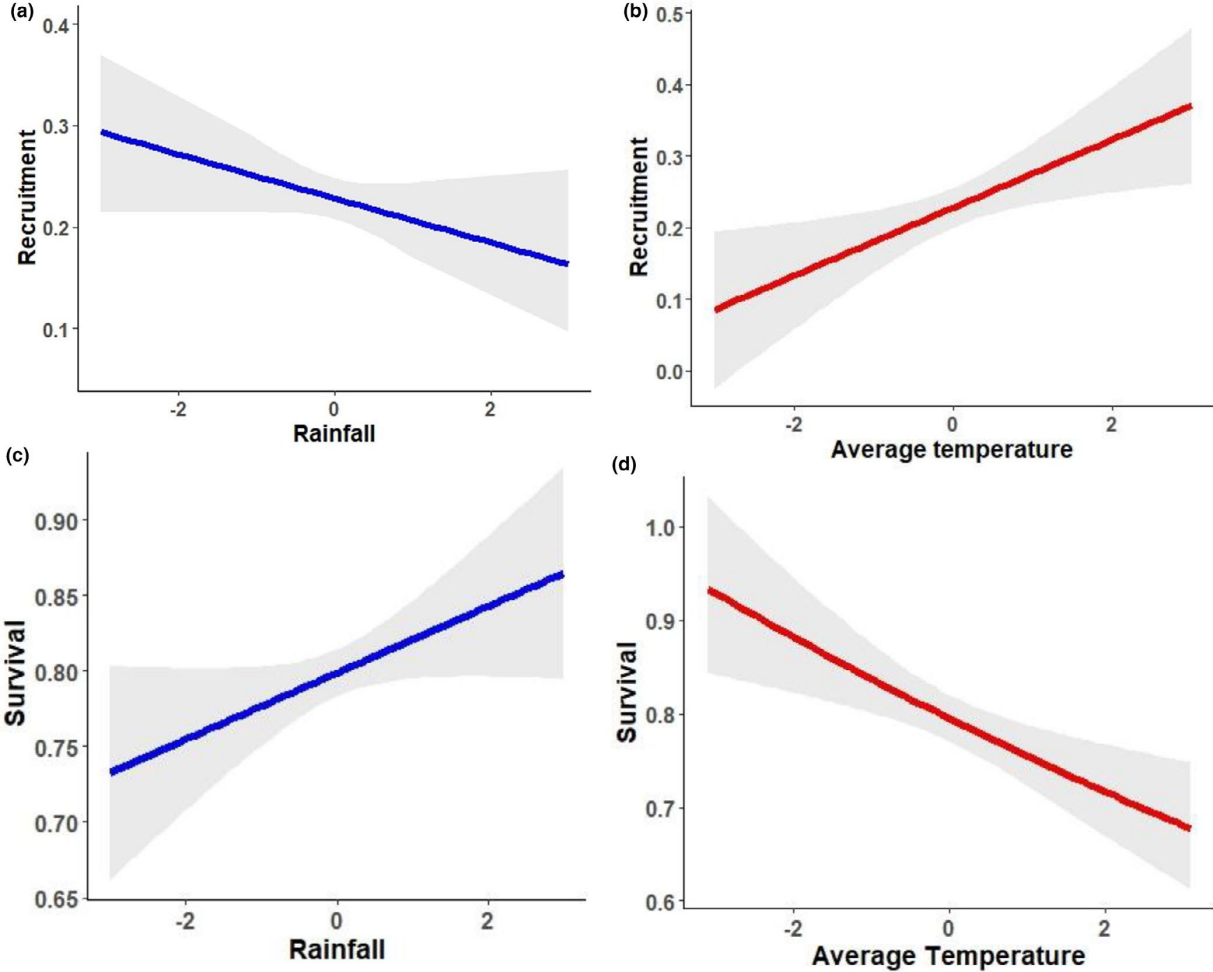
Effects of climatic variables (*z*‐transformed) on survival and recruitment of brush mice (*Peromyscus boylii*) at Camp Roberts, California based on the most parsimonious model that included the given covariate for each demographic rate; (a) relationship between rainfall and recruitment (*f*); (b) relationship between temperature and recruitment (*f*); (c) relationship between rainfall and survival (*φ*); (d) relationship between temperature and survival (*φ*)

The most parsimonious model for single covariate effects exhibited 92% of the AICc weight and included the effect of variation in rainfall on *φ* and average temperature on *f* (Table 2c). Although the top five models for *φ* included only variation in rainfall, rainfall with a one‐season lag, and average temperature, all other variables except El Niño had significant effects on *φ* (Table 3). Rainfall and variation in rainfall with a one‐season lag had positive effects on *φ*, while average temperature, variation in temperature, variation in rainfall, and rainfall with a one‐season lag had negative effects on *φ* (Table 3; Figure 4c, d).

**TABLE 3:** The effect of climatic covariates on apparent survival (φ), recruitment (*f*), and realized population growth rate (λ) of brush mice (*Peromyscus boylii*) in a coastal‐central California mixed‐oak woodland

Climate covariate	Demographic parameter		
	Survival (φ)	Recruitment (*f*)	Growth rate (λ)
Temp_avg	**−0.11 ± 0.05**	**0.24 ± 0.05**	0.007 ± 0.009
Temp_cv	**−0.075 ± 0.03**	**0.069 ± 0.037**	−0.002 ± 0.003
Rain_sum	**0.17 ± 0.04**	**−0.165 ± 0.045**	**0.026 ± 0.004**
Rain_sum_onelag	**−0.12 ± 0.03**	**0.185 ± 0.031**	**0.074 ± 0.005**
Rain_cv	**−0.13 ± 0.03**	**0.142 ± 0.034**	**−0.011 ± 0.005**
Rain_cv_onelag	**0.1 ± 0.04**	**−0.090 ± 0.034**	**−0.015 ± 0.004**
El Niño	−0.03 ± 0.02	−0.003 ± 0.02	0.009 ± 0.01

We report the estimate of slope parameters (β ± SE) based on the most parsimonious model that included a given covariate for each demographic rate, regardless of model structure for other demographic rates. Estimates in bold indicate that 95% CI for β do not include zero.

Although the top five most parsimonious models for *f* included average temperature, rainfall, and variation in rainfall with a one‐season lag, all other variables except El Niño had a significant effect on *f*. Average temperature, variation in temperature, variation in rainfall, and rainfall with a one‐season lag had a positive effect on recruitment, while rainfall, and variation in rainfall with a one‐season lag had negative effects on *f* (Table 3; Figure 4a, b). The effect of climatic covariates on *λ* was similar to the effect of climatic covariates on *φ*. However, the overall strength of these effects was lower for *λ* than for *φ*. Rainfall and variation in rainfall with a one‐season lag had a positive effect on *λ*, while average temperature and variation in temperature had a negative effect on *λ* (Table 3). Models did not support an interactive effect of season and temperature or season and rainfall on either *f*, *φ*, or *λ*. Rainfall and temperature showed high negative correlation (*r =* −0.81, *p* < .001).

## 4 DISCUSSION

### 4.1 Effect of rainfall on Φ

Although the brush mouse population was stable throughout the study period, there was substantial temporal variation in survival and recruitment, and we posit that these fluctuations were driven largely by climatic factors. All climatic variables had a strong and significant effect on both *φ* and *f*, except for El Niño events. For example, single‐covariate models showed a positive effect of rainfall on *φ* (*β* = 0.17 ± 0.04; Table 3), and fluctuations in *φ* were highly correlated with rainfall (*r = *0.44, *p =* .005), in agreement with our hypotheses. The effects of precipitation on small mammal population vital rates in semi‐arid systems are generally well studied (Brown & Ernest, 2002; Heske et al., 1994; Letnic & Dickman, 2005; Lima et al., 2002; Meserve et al., 2003), and a widely accepted hypothesis posits that higher precipitation in semi‐arid systems increases primary production, leading to an increase in survival of small mammal populations (Heske et al., 1994; Letnic & Dickman, 2005; Lima et al., 2002; Shenbrot & Krasnov, 2001; Srivathsa et al., 2019; Tietje et al., 2018; Yates et al., 2002). Studies have associated increased rodent densities with higher precipitation in various habitats (Brown & Ernest, 2002; Kuenzi et al., 2007; Meserve et al., 1995). Because the brush mouse is a shrub‐habitat specialist, its survival will be especially affected by precipitation and its effect on primary production, which directly impacts food availability and refuge from weather and predators (Baker, 1968; Bradley & Schmidly, 1999; Kalcounis‐Rüppell & Millar, 2002). Emphasizing the crucial importance of vegetative cover for this species, a wildfire that appreciably consumed brush cover led to a 90% decrease in brush mouse populations (Brehme et al., 2011). As an omnivore, the brush mouse consumes fruits and seeds of a wide variety of plant species as well as insects, which are positively affected by rainfall in resource‐restricted semi‐arid environments (Fuentes & Campusano, 1985; Yang et al., 2011). Rainfall typically increases the amount of understory shrub and chaparral cover, suggesting that the ability of the brush mouse to find food, survive in inclement weather, and evade predators will increase with higher precipitation and lead to higher survival rates, through both lower mortality and higher emigration.

### 4.2 Effect of rainfall on *f*

In contrast to its effect on survival, covariate models showed a negative influence of rainfall on recruitment (*β* = −0.165 ± 0.045; Table 3). This negative effect may be due to increased energetic costs incurred by reproductive females, or the destruction of food stores and nesting sites used by reproductive females during times of heavy precipitation. Although this is contrary to the general expectation that typically shows higher recruitment in response to increasing precipitation (e.g., see Previtali et al., 2009; Shenbrot & Krasnov, 2001; Srivathsa et al., 2019; Thibault et al., 2010; Tietje et al., 2018), the effect of precipitation is not necessarily linear or simple. Extreme rainfall events can lead to catastrophic declines of small mammals, attributed to the demolishment of food stores and nesting areas (Chaudhary et al., 2021; Rolland et al., 2021; Thibault & Brown, 2008; Valone & Brown, 1995), especially when located underground or just above the ground (shrubs or logs), habitats typically used by brush mice for nesting. High rainfall, especially during cold periods, can also cause abrupt declines in mouse populations (Calisher et al., 2005; de Villafane & Bonaventura, 1987; Garsd & Howard, 1981; Lewellen & Vessey, 1998; Mills, 2005; Mills et al., 1992). Such unfavorable climatic conditions affect reproduction through mortality from direct exposure or lack of access to food or shelter. Furthermore, exposure to heavy rainfall, and the colder temperatures that usually accompany rainstorms, can result in populations with smaller and fewer litters, possibly due to the higher energetic costs of maintaining a suitable microclimate for the litter (Myers et al., 1985).

While rainfall negatively affected recruitment, rainfall with a one‐season lag had a positive effect on recruitment. In these systems, precipitation typically drives plant productivity, but foliage growth and seed production might occur over several months, potentially leading to the lag effect we detected in terms of benefits of rainfall for rodent reproduction, immigration, and population growth rate (Dickman et al., 1999; Greenville et al., 2016). Thus, the direct effect of rainfall decreased brush mouse recruitment, but the consequent effects of rainfall on primary productivity seemed beneficial to recruitment.

### 4.3 Effect of temperature on *φ* and *f*

Temperature was negatively correlated with *φ* (*β* = −0.11 ± 0.05; Table 3). We suspect this is because foraging and juvenile dispersal would become energetically costly and difficult to perform with higher temperatures, potentially leading to decreased *φ* (Bradley & Schmidly, 1999). In contrast to our hypotheses, average temperature had a significant positive effect on *f* (*β* = 0.24 ± 0.05; Table 3)—similar to the positive effect of average temperature on *f* of the California pocket mouse (*Chaetodipus californicus*—Chaudhary et al., 2021) and the big‐eared woodrat (*Neotoma macrotis*—Rolland et al., 2021) in the same study area. This could be attributed to two plausible reasons: (1) *f* could increase with warmer temperatures because brush mice can breed more frequently, especially during winter (Bradley & Schmidly, 1999; California Department of Fish & Wildlife, 2014), and (2) higher temperatures in spring and early summer increase primary productivity, allowing female mice to expend less energetic costs for foraging, and ensuring a more favorable microclimate for litters due to an increase in vegetative cover for nests, which are typically found in trees and shrubs (Kalcounis‐Rüppell, 2002). Season and temperature are known to have interactive and sometimes contradictory effects on small mammal populations (Luis et al., 2010), however, contrary to our hypotheses, our data did not support an interaction between season and temperature on brush mouse vital rates.

Although studies have shown that El Niño events can have significant effects on small mammal survival and recruitment (Shenbrot & Krasnov, 2001), El Niño events did not have an effect on survival, recruitment, or growth rates of brush mice on our study area. This could be because, out of the last 23 El Niño events that were predicted for this study area, the majority did not occur (Null, 2015).

### 4.4 Trade‐offs between *φ* and *f* in the maintenance of population stability

Brush mouse population growth rate (*λ*) was stable throughout the study (*λ* = 0.993 ± 0.032). While *φ* was positively affected by rainfall and negatively affected by temperature, *f* was positively affected by temperature and negatively affected by rainfall (Figure 4). These contrasting effects of climatic factors on vital rates suggest that the brush mouse employs alternating strategies in maintaining population stability, dependent on climatic conditions. Additionally, *φ* and *f* estimates were highly negatively correlated (*r =* −0.61, *p =* .002), implying a trade‐off between *φ* and *f*. During hotter, drier seasons, brush mouse populations invest in higher *f* as a means of maintaining the population, whereas during cooler, wetter seasons, brush mice invest in higher *φ* to maintain population *λ*. Considered together, the brush mouse appears to adopt a combination of coping mechanisms to ensure population stability. Moreover, the contribution of *φ* to *λ* was consistently higher than *f*, as inferred from the proportional contribution parameter (Nichols & Hines, 2002; Schorr, 2012). The estimate of *γ* averaged 0.82; that is, on average, 82% of individuals in the current month are individuals that survived from the previous month. Although small mammals are typically *r*‐selected, where population increase is fueled more by *f* than *φ* (Heppell et al., 2000; Oli & Dobson, 2003), the brush mouse seems to be less r‐selected than other small mammal species (Schorr, 2012) due to the relatively higher contribution of *φ* to *λ* for this species. The relatively higher contribution of *φ* toward *λ* was supported by the overall climatic effects on vital rates. The effects of rainfall and temperature were similar for *φ* and *λ* (a positive effect of rainfall, and a negative effect of temperature on both parameters). However, these effects were reversed for *f* (a negative effect of rainfall, a positive effect of temperature). The relatively higher contribution of *φ* toward *λ* than what is typically observed for small mammals is also supported by the fact that the brush mouse prioritizes *φ* as a coping strategy and does not rely primarily on *f* to ensure population stability.

### 4.5 Climate change

Climate change forecast models suggest that climatic conditions will likely become increasingly hotter and drier, with long dry periods punctuated by dramatic rainfall events (Gian‐Reto et al., 2002; IPCC, 2018). Our study results indicate that these climatic changes will have differing effects on brush mouse vital rates. As we predicted, survival of the population of brush mice on our study area decreased during hot, dry summer conditions. Increasingly, hotter and drier conditions may thus not bode well for brush mice survival, especially because dry conditions typically worsen the effect of increased temperatures. Our results suggest that brush mouse survival might decrease with climatic change, leading to population decline. These changes might be especially detrimental to brush mouse populations as survival makes a relatively higher contribution toward population growth rate than recruitment. Consequently, recruitment, which increased during hot, dry periods, may become the more dominant coping strategy in maintaining population stability. However, dramatic rainfall events might have a negative effect on brush mouse recruitment, suggesting that an increase in recruitment due to hotter and drier conditions might be tempered by more frequent, heavy rainfall. Brush mice are also particularly dependent on ground and vegetative cover, mesic environments, and shrubs and trees for foraging and nesting (California Department of Fish & Wildlife, 2014). Since primary productivity is negatively affected by an increase in dry conditions, climate change associated with more frequent and more intense droughts could be detrimental to structural habitats that are important for the species.

Climate models also predict an increase in the frequency and magnitude of wildfires, especially in southern California and the western Sierras (Westerling & Bryant, 2008), which are extremely detrimental to brush mouse populations (Brehme et al., 2011). In addition to these, the invasive pathogen *Phytophthora ramorum*, which causes Sudden Oak Death in tanoak and other oak species (McPherson et al., 2010), is one of the pathogens that may become more widespread and harder to control with changing climatic conditions (Brown & Allen‐Diaz, 2006). The spread of this pathogen could be especially problematic for brush mice, which rely on tanoak acorns (Reid et al., 2013) and coast live oak acorns (Kalcounis‐Rüppell, 2002), as a primary food source. However, the varying coping strategies employed by the brush mouse in response to varying environmental conditions may serve as a mechanism by which the species maintains resilience in the face of changing climatic conditions, although brush mouse populations might not be able to withstand other changes such as larger and more frequent wildfires.

## 5 CONCLUSIONS

Our study was the first to apply Pradel's temporal symmetry models to brush mouse populations and demonstrated the implementation of a modern demographic modeling framework to quantify the effect of *f* and *φ* on population *λ* for a brush mouse population. Although brush mouse population dynamics were influenced by localized climatic effects, the overall population size remained stable and appeared resilient to annual and multiannual fluctuations. This brush mouse population utilized a trade‐off between survival and recruitment to maintain population stability, using high *f* during warmer, drier seasons and high *φ* during cooler, wetter seasons to sustain population size. These varying coping strategies may serve as a mechanism by which the species maintains resilience in the face of climate change. Brush mice play an important role in ecosystem processes, as they are extremely important prey for a large variety of terrestrial and avian predators, including the federally threatened Mexican spotted owl (Boyett, 2001). Brush mouse are also reservoir hosts for the hantavirus, raising important health concerns associated with rates of spread. Future investigations are needed to build on our findings and examine other aspects such as predation, intraguild competition, and the potential influence of spatial attributes on population dynamics, factors that, in addition to our results, will likely have implications for the management and conservation of the species.

## CONFLICT OF INTEREST

The authors declare no competing interests.

## AUTHOR CONTRIBUTION

**Ann Polyakov:** Data curation (supporting); Formal analysis (lead); Investigation (equal); Methodology (supporting); Visualization (lead); Writing‐original draft (lead); Writing‐review & editing (equal). **William D. Tietje:** Conceptualization (lead); Data curation (lead); Investigation (lead); Methodology (supporting); Writing‐review & editing (equal). **Arjun Srivathsa:** Conceptualization (supporting); Formal analysis (supporting); Investigation (supporting); Methodology (supporting); Software (supporting); Visualization (supporting); Writing‐review & editing (supporting). **Virginie Rolland:** Conceptualization (supporting); Formal analysis (supporting); Investigation (supporting); Methodology (supporting); Visualization (supporting); Writing‐review & editing (supporting). **James E. Hines:** Formal analysis (supporting); Methodology (supporting); Resources (equal); Software (lead); Writing‐review & editing (supporting). **Madan K. Oli:** Conceptualization (supporting); Formal analysis (equal); Methodology (equal); Software (lead); Validation (equal); Writing‐review & editing (supporting).

## Supporting information

Table S1

## Data Availability

The dataset and code for this study are available within the Harvard Dataverse repository at https://doi.org/10.7910/DVN/I6DZZH).

## References

[ece37997-bib-0001] Abbott, K. D., Ksiazek, T. G., & Mills, J. N. (1999). Long‐term hantavirus persistence in rodent populations in central Arizona. Emerging Infectious Diseases, 5(1), 102–112. 10.3201/eid0501.990112 10081677 PMC2627700

[ece37997-bib-0002] Andreo, V., Lima, M., Provensal, C., Priotto, J., & Polop, J. (2009). Population dynamics of two rodent species in agro‐ecosystems of central Argentina: Intra‐specific competition, land‐use, and climate effects. Population Ecology, 51(2), 297–306. 10.1007/s10144-008-0123-3

[ece37997-bib-0003] Arnason, A. N., & Schwarz, C. J. (1995). POPAN‐4: Enhancements to a system for the analysis of mark‐recapture data from open populations. Journal of Applied Statistics, 22(5–6), 785–800. 10.1080/02664769524621

[ece37997-bib-0004] Baker, R. H. (1968). Habitats and distribution. In J. A. King (Ed.), Biology of Peromyscus (Rodentia) (pp. 98–126). Special Publication No. 2, American Society of Mammalogists.

[ece37997-bib-0005] Boyett, W. D. (2001). Habitat relations of rodents in the Hualapai Mountains of northwestern Arizona. Doctoral Dissertation. University of Wisconsin‐Oshkosh.

[ece37997-bib-0006] Bradley, R. D., & Schmidly, D. J. (1999). Brush mouse: *Peromyscus boylii* . In D. E. Wilson, & S. Ruff (Eds.), The Smithsonian book of North American mammals (pp. 564–565). Smithsonian Institution Press.

[ece37997-bib-0007] Brehme, C. S., Clark, D. R., Rochester, C. J., & Fisher, R. N. (2011). Wildfires alter rodent community structure across four vegetation types in southern California, USA. Fire Ecology, 7(2), 81–98. 10.4996/fireecology.0702081

[ece37997-bib-0008] Brown, J. H., & Ernest, S. M. (2002). Rain and rodents: Complex dynamics of desert consumers: Although water is primary limiting resource in desert ecosystems, relationship between rodent population dynamics and precipitation is complex and nonlinear. BioScience, 52(11), 979–987.

[ece37997-bib-0009] Brown, L. B., & Allen‐Diaz, B. (2006). Forecasting the future of coast live oak forests in the face of sudden oak death (pp. 179–180). Gen. Tech. Rep. PSW‐GTR‐196. Pacific Southwest Research Station, Forest Service, US Department of Agriculture.

[ece37997-bib-0010] Burnham, K. P., & Anderson, D. R. (2002). Information and likelihood theory: A basis for model selection and inference. In K. P. Burnham, & D. R. Anderson (Eds.), Model selection and multimodel inference: A practical information‐theoretic approach (pp. 49–97). New York, NY, USA: Springer‐Verlag.

[ece37997-bib-0011] California Department of Fish and Wildlife (2014). California Interagency Wildlife Task Group. CWHR Version 9.0 Personal Computer Program.

[ece37997-bib-0012] Calisher, C. H., Mills, J. N., Sweeney, W. P., Root, J. J., Reeder, S. A., Jentes, E. S., & Beaty, B. J. (2005). Population dynamics of a diverse rodent assemblage in mixed grass‐shrub habitat, southeastern Colorado. Journal of Wildlife Diseases, 41(1), 12–28. 15827207 10.7589/0090-3558-41.1.12

[ece37997-bib-0013] Chaudhary, V., Tietje, W. D., Polyakov, A. Y., Rolland, V., & Oli, M. K. (2021). Factors driving California pocket mice (*Chaetodipus californicus*) population dynamics. Journal of Mammalogy, 1–12.

[ece37997-bib-0014] Chen, L., Wang, G., Wan, X., & Liu, W. (2015). Complex and nonlinear effects of weather and density on the demography of small herbivorous mammals. Basic and Applied Ecology, 16(2), 172–179. 10.1016/j.baae.2014.12.002

[ece37997-bib-0015] De Villafane, G., & Bonaventura, S. M. (1987). Ecological studies in crop fields of the endemic area of Argentine hemorrhagic fever. *Calomys musculinus* movements in relation to habitat and abundance. Mammalia, 51(2), 233–248.

[ece37997-bib-0016] Dickman, C. R., Mahon, P. S., Masters, P., & Gibson, D. F. (1999). Long‐term dynamics of rodent populations in arid Australia: The influence of rainfall. Wildlife Research, 26(4), 389–403. 10.1071/WR97057

[ece37997-bib-0017] Facka, A. N., Roemer, G. W., Mathis, V. L., Kam, M., & Geffen, E. (2010). Drought leads to collapse of black‐tailed prairie dog populations reintroduced to the Chihuahuan Desert. The Journal of Wildlife Management, 74(8), 1752–1762. 10.2193/2009-208

[ece37997-bib-0018] Fuentes, E. R., & Campusano, C. (1985). Pest outbreaks and rainfall in the semi‐arid region of Chile. Journal of Arid Environments, 8(1), 67–72. 10.1016/S0140-1963(18)31338-7

[ece37997-bib-0019] Garsd, A., & Howard, W. E. (1981). A 19‐year study of microtine population fluctuations using time‐series analysis. Ecology, 62(4), 930–937. 10.2307/1936991

[ece37997-bib-0020] Gian‐Reto, W., Post, E., Convey, P., Menzel, A., Parmesan, C., Beebee, T. J. C., & Bairlein, F. (2002). Ecological responses to recent climate change. Nature, 416(6879), 389–395. 11919621 10.1038/416389a

[ece37997-bib-0021] Gillespie, S. C., Van Vuren, D. H., Kelt, D. A., Eadie, J. M., & Anderson, D. W. (2008). Dynamics of rodent populations in semiarid habitats in Lassen County, California. Western North American Naturalist, 68(1), 76–83.

[ece37997-bib-0022] Gottesman, A. B., Krausman, P. R., Morrison, M. L., & Petryszyn, Y. (2004). Movements and home range of brush mice. The Southwestern Naturalist, 49(2), 289–295.

[ece37997-bib-0023] Greenville, A. C., Wardle, G. M., Nguyen, V., & Dickman, C. R. (2016). Population dynamics of desert mammals: Similarities and contrasts within a multispecies assemblage. Ecosphere, 7(5), e01343. 10.1002/ecs2.1343

[ece37997-bib-0024] Gutiérrez, J. R., Meserve, P. L., Kelt, D. A., Engilis, A. Jr, Previtali, M. A., Milstead, W. B., & Jaksic, F. M. (2010). Long‐term research in Bosque Fray Jorge National Park: Twenty years studying the role of biotic and abiotic factors in a Chilean semiarid scrubland. Revista Chilena De Historia Natural, 83(1), 69–98. 10.4067/S0716-078X2010000100005

[ece37997-bib-0025] Heppell, S. S., Caswell, H., & Crowder, L. B. (2000). Life histories and elasticity patterns: Perturbation analysis for species with minimal demographic data. Ecology, 81(3), 654–665.

[ece37997-bib-0026] Heske, E. J., Brown, J. H., & Mistry, S. (1994). Long‐term experimental study of a Chihuahuan Desert rodent community: 13 years of competition. Ecology, 75(2), 438–445. 10.2307/1939547

[ece37997-bib-0027] IPCC (2018). Summary for Policymakers. In V. Masson‐Delmotte, P. Zhai, H.‐O. Pörtner, D. Roberts, J. Skea, P. R. Shukla, A. Pirani, W. Moufouma‐Okia, C. Péan, R. Pidcock, S. Connors, J. B. R. Matthews, Y. Chen, X. Zhou, M. I. Gomis, E. Lonnoy, T. Maycock, M. Tignor, & T. Waterfield (Eds.), Global Warming of 1.5°C. An IPCC Special Report on the impacts of global warming of 1.5°C above pre‐industrial levels and related global greenhouse gas emission pathways, in the context of strengthening the global response to the threat of climate change, sustainable development, and efforts to eradicate poverty (pp. 32). World Meteorological Organization.

[ece37997-bib-0029] Kalcounis‐Rüppell, M. C. (2002). Breeding systems, habitat overlap, and activity patterns of monogamous and promiscuous mating in *Peromyscus californicus* and *Peromyscus boylii* . Doctoral Dissertation. The University of Western Ontario.

[ece37997-bib-0030] Kalcounis‐Rüppell, M. C., & Millar, J. S. (2002). Partitioning of space, food, and time by syntopic *Peromyscus boylii* and *Peromyscus californicus* . Journal of Mammalogy, 83(2), 614–625.

[ece37997-bib-0031] Knapp, A. K., Beier, C., Briske, D. D., Classen, A. T., Luo, Y., Reichstein, M., Smith, M. D., Smith, S. D., Bell, J. E., Fay, P. A., Heisler, J. L., Leavitt, S. W., Sherry, R., Smith, B., & Weng, E. (2008). Consequences of more extreme precipitation regimes for terrestrial ecosystems. BioScience, 58(9), 811–821. 10.1641/B580908

[ece37997-bib-0032] Kuenzi, A. J., Morrison, M. L., Madhav, N. K., & Mills, J. N. (2007). Brush mouse (*Peromyscus boylii*) population dynamics and hantavirus infection during a warm, drought period in southern Arizona. Journal of Wildlife Diseases, 43(4), 675–683. 10.7589/0090-3558-43.4.675 17984263

[ece37997-bib-0033] Kuenzi, A. J., Morrison, M. L., Swann, D. E., Hardy, P. C., & Downard, G. T. (1999). A longitudinal study of Sin Nombre virus prevalence in rodents, southeastern Arizona. Emerging Infectious Diseases, 5(1), 113. 10.3201/eid0501.990113 10081678 PMC2627683

[ece37997-bib-0028] L., J. L. (2013). RMark: an R interface for analysis of capture‐recapture data with MARK. AFSC Processed Rep. 2013‐01, Alaska Fisheries Science Center, National Oceanic and Atmospheric Administration, National Marine Fisheries Service. http://www.afsc.noaa.gov/Publications/ProcRpt/PR2013‐01.pdf

[ece37997-bib-0034] Letnic, M., & Dickman, C. R. (2005). The responses of small mammals to patches regenerating after fire and rainfall in the Simpson Desert, central Australia. Australian Ecology, 30(1), 24–39.

[ece37997-bib-0035] Lewellen, R. H., & Vessey, S. H. (1998). The effect of density dependence and weather on population size of a polyvoltine species. Ecological Monographs, 68(4), 571–594.

[ece37997-bib-0036] Lima, M., Stenseth, N. C., & Jaksic, F. M. (2002). Population dynamics of S. American rodent: Seasonal structure interacting with climate, density dependence and predator effects. Proceedings of the Royal Society of London. Series B: Biological Sciences, 269(1509), 2579–2586. 12573073 10.1098/rspb.2002.2142 PMC1691189

[ece37997-bib-0037] Luis, A. D., Douglass, R. J., Mills, J. N., & Bjørnstad, O. N. (2010). The effect of seasonality, density and climate on the population dynamics of Montana deer mice, important reservoir hosts for Sin Nombre hantavirus. Journal of Animal Ecology, 79(2), 462–470. 10.1111/j.1365-2656.2009.01646.x 20015212

[ece37997-bib-0038] McPherson, B. A., Mori, S. R., Wood, D. L., Kelly, M., Storer, A. J., Svihra, P., & Standiford, R. B. (2010). Responses of oaks and tanoaks to sudden oak death pathogen in two coastal California forests. Forest Ecology and Management, 259(12), 2248–2255.

[ece37997-bib-0039] Meserve, P. L., Kelt, D. A., Milstead, W. B., & Gutiérrez, J. R. (2003). Thirteen years of shifting top‐down and bottom‐up control. BioScience, 53(7), 633–646.

[ece37997-bib-0040] Meserve, P. L., Yunger, J. A., Gutiérrez, J. R., Contreras, L. C., Milstead, W. B., Lang, B. K., Cramer, K. L., Herrera, S., Lagos, V. O., Silva, S. I., & Tabilo, E. L. (1995). Heterogeneous responses of small mammals to an El Niño Southern Oscillation event in northcentral semiarid Chile and the importance of ecological scale. Journal of Mammalogy, 76(2), 580–595.

[ece37997-bib-0041] Mills, J. N. (2005). Regulation of rodent‐borne viruses in the natural host: implications for human disease. Infectious diseases from nature: mechanisms of viral emergence and persistence (pp. 45–57). Springer. 10.1007/3-211-29981-5_5 16355867

[ece37997-bib-0042] Mills, J. N., Ellis, B. A., McKee, K. T. Jr, Calderon, G. E., Maiztegui, J. I., Nelson, G. O., Ksiazek, T. G., Peters, C. J., & Childs, J. E. (1992). A Longitudinal Study of Junin Virus Activity in the Rodent Reservoir of Argentine Hemorrhagic Fever. The American Journal of Tropical Medicine and Hygiene, 47(6), 749–763. 1335214 10.4269/ajtmh.1992.47.749

[ece37997-bib-0043] Morrison, M. L., Kuenzi, A. J., Brown, C. F., & Swann, D. E. (2002). Habitat use and abundance trends of rodents in southeastern Arizona. The Southwestern Naturalist, 47(4), 519–526. 10.2307/3672654

[ece37997-bib-0044] Myers, P., Master, L. L., & Garrett, R. A. (1985). Ambient temperature and rainfall: An effect on sex ratio and litter size in deer mice. Journal of Mammalogy, 66(2), 289–298. 10.2307/1381241

[ece37997-bib-0045] National Oceanic and Atmospheric Administration (2016). Climate Data Online. https://www.ncdc.noaa.gov/cdo‐web/

[ece37997-bib-0046] Nichols, J. D., & Hines, J. E. (2002). Approaches for the direct estimation of u, and demographic contributions to u. Journal of Applied Statistics, 29(1–4), 539–568.

[ece37997-bib-0047] Nichols, J. D., Hines, J. E., Lebreton, J. D., & Pradel, R. (2000). Estimation of contributions to population growth: A reverse‐time capture–recapture approach. Ecology, 81(12), 3362–3376.

[ece37997-bib-0048] Null, J. (2015). Golden Gate Weather Services – the misconceptions of El Niño. http://ggweather.com/enso/enso_myths.htm

[ece37997-bib-0049] Oli, M. K. (2004). The fast–slow continuum and mammalian life‐history patterns: An empirical evaluation. Basic and Applied Ecology, 5(5), 449–463. 10.1016/j.baae.2004.06.002

[ece37997-bib-0050] Oli, M. K., & Dobson, F. S. (2003). The relative importance of life‐history variables to population growth rate in mammals: Cole’s prediction revisited. The American Naturalist, 161(3), 422–440. 10.1086/367591 12699222

[ece37997-bib-0051] Oli, M., & Dobson, F. (2005). Generation Time Elasticity Patterns and Mammalian Life Histories: A Reply to Gaillard et al The American Naturalist, 166(1), 124–128. 10.1086/430332 15937795

[ece37997-bib-0052] Peel, M. C., Finlayson, B. L., & McMahon, T. A. (2007). Updated world map of the Köppen‐Geiger climate classification. Hydrology and Earth System Sciences Discussions, 4(2), 439–473.

[ece37997-bib-0053] Pradel, R. (1996). Utilization of capture‐mark‐recapture for the study of recruitment and population growth rate. Biometrics, 52(2), 703–709. 10.2307/2532908

[ece37997-bib-0054] Previtali, M. A., Lima, M., Meserve, P. L., Kelt, D. A., & Gutiérrez, J. R. (2009). Population dynamics of two sympatric rodents in a variable environment: Rainfall, resource availability, and predation. Ecology, 90(7), 1996–2006. 10.1890/08-0405.1 19694146

[ece37997-bib-0055] R Core Team (2019). R: A language and environment for statistical computing. R Foundation for Statistical Computing. http://www.R‐project.org

[ece37997-bib-0056] Reid, R. E., Greenwald, E. N., Wang, Y., & Wilmers, C. C. (2013). Dietary niche partitioning by sympatric Peromyscus boylii and P. californicus in a mixed evergreen forest. Journal of Mammalogy, 94(6), 1248–1257.

[ece37997-bib-0057] Rolland, V., Tietje, W. D., Polyakov, A. Y., Chaudhary, V., & Oli, M. K. (2021). Climatic factors and population demography in big‐eared woodrat, *Neotoma macrotis* . Journal of Mammalogy, 102(3), 731–742.

[ece37997-bib-0058] Schorr, R. A. (2012). Using a temporal symmetry model to assess population change and recruitment in the Preble's meadow jumping mouse (*Zapus hudsonius preblei*). Journal of Mammalogy, 93(5), 1273–1282.

[ece37997-bib-0059] Schwarz, C. J., & Arnason, A. N. (1996). A general methodology for the analysis of capture‐recapture experiments in open populations. Biometrics, 52(3), 860–873. 10.2307/2533048

[ece37997-bib-0060] Shenbrot, G., & Krasnov, B. (2001). Rodents in desert environments: Is density dynamics really correlated with annual rainfall fluctuations. Ecology of desert environments. Festschrift for professor JL Cloudsley‐Thompson (pp. 405–421). Scientific Publishers.

[ece37997-bib-0061] Sikes, R. S. (2016). 2016 Guidelines of the American Society of Mammalogists for the use of wild mammals in research and education:. Journal of Mammalogy, 97(3), 663–688. 10.1093/jmammal/gyw078 29692469 PMC5909806

[ece37997-bib-0062] Srivathsa, A., Tietje, W., Rolland, V., Polyakov, A., & Oli, M. K. (2019). Climatic drivers of pinyon mouse *Peromyscus truei* population dynamics in a resource‐restricted environment. Population Ecology, 61(1), 122–131.

[ece37997-bib-0063] Thibault, K., & Brown, J. (2008). Impact of an extreme climatic event on community assembly. Proceedings of the National Academy of Sciences of the United States of America, 105(9), 3410–3415. 10.1073/pnas.0712282105 18303115 PMC2265133

[ece37997-bib-0064] Thibault, K., Ernest, S., White, E., Brown, J., & Goheen, J. (2010). Long‐term insights into the influence of precipitation on community dynamics in desert rodents. Journal of Mammalogy, 91(4), 787–797. 10.1644/09-MAMM-S-142.1

[ece37997-bib-0065] Tietje, W. D., Polyakov, A. Y., Rolland, V., Hines, J. E., & Oli, M. K. (2018). Climatic influences on demography of the California mouse (*Peromyscus californicus*) in semiarid oak woodland. Journal of Mammalogy, 99(5), 1149–1158. 10.1093/jmammal/gyy089

[ece37997-bib-0066] Tietje, W. D., Vreeland, J. K., Siepel, N., & Dockter, J. L. (1997). Relative abundance and habitat associations of vertebrates in oak woodlands in coastal‐central California. U.S. Forest Service, General Technical Report PSW‐GTR, 160, 391–400.

[ece37997-bib-0067] Turchin, P. (2003). Complex population dynamics: A theoretical/empirical synthesis. Monographs in population biology (pp. 35). Princeton University Press.

[ece37997-bib-0068] Valone, T., & Brown, J. (1995). Effects of competition, colonization, and extinction on rodent species diversity. Science, 267(5199), 880–883. 7846530 10.1126/science.7846530

[ece37997-bib-0069] Westerling, A., & Bryant, L. (2008). Climate change and wildfire in California. Climatic Change, 87(1), 231–249. 10.1007/s10584-007-9363-z

[ece37997-bib-0070] White, G., & Burnham, K. (1999). Program MARK: Survival estimation from populations of marked animals. Bird Study, 46, 120–139. 10.1080/00063659909477239

[ece37997-bib-0071] Williams, B., Nichols, J. D., & Conroy, M. J. (2002). Analysis and management of animal populations: Modeling, estimation, and decision making. Academic Press.

[ece37997-bib-0072] Yang, H., Li, Y., Wu, M., Zhang, Z., Li, L., & Wan, S. (2011). Plant community responses to nitrogen addition and increased precipitation: The importance of water availability and species traits. Global Change Biology, 17(9), 2936–2944. 10.1111/j.1365-2486.2011.02423.x

[ece37997-bib-0073] Yates, T. L., Mills, J. N., Parmenter, C. A., Ksiazek, T. G., Parmenter, R. R., Vande castle, J. R., Calisher, C. H., Nichol, S. T., Abbott, K. D., Young, J. C., Morrison, M. L., Beaty, B. J., Dunnum, J. L., Baker, R. J., Salazar‐bravo, J., & Peters, C. J. (2002). The Ecology and Evolutionary History of an Emergent Disease: Hantavirus Pulmonary Syndrome. BioScience, 52(11), 989–998.

